# Neurofilament Light Chain Is a Promising Biomarker in Alcohol Dependence

**DOI:** 10.3389/fpsyt.2021.754969

**Published:** 2021-11-18

**Authors:** Yanfei Li, Ranran Duan, Zhe Gong, Lijun Jing, Tian Zhang, Yong Zhang, Yanjie Jia

**Affiliations:** ^1^Department of Neurology, First Affiliated Hospital of Zhengzhou University, Zhengzhou, China; ^2^Department of Rehabilitation, First Affiliated Hospital of Zhengzhou University, Zhengzhou, China; ^3^Department of Magnetic Resonance Imaging, First Affiliated Hospital of Zhengzhou University, Zhengzhou, China

**Keywords:** NLRP3, NfL, alcohol dependence, biomarker, white matter lesions, gray matter volume, neuropsychological assessment

## Abstract

**Background:** Alcohol dependence, a global public health problem, leads to structural and functional damage in the brain. Alcohol dependence patients present complex and varied clinical manifestations and live with general complaints existing in contemporary society, making most people with alcohol dependence hard to identify. Therefore, it is important to find potential biomarkers for the diagnosis and evaluation of alcohol dependence. In the study, we explored potential biomarkers for the diagnosis and monitoring of diseases and evaluated brain structural changes in alcohol dependence patients.

**Methods:** Enzyme-linked immunosorbent assay (ELSA) was employed to detect the expression of serum nucleotide-binding oligomerization domain containing 3 (NLRP3) and single-molecule array (Simoa) assay was used to detect the expression of serum neurofilament light (NfL) in 50 alcohol dependence patients and 50 controls with no drinking history. Alcohol consumption was measured by standard drinks. Neuropsychological assessments, including the Montreal cognitive assessment (MoCA), Pittsburgh sleep quality index (PSQI), generalized anxiety disorder (GAD-7), and patient health questionnaire-9 (PHQ-9), were conducted to evaluate cognitive function and psychological state. The degree of white matter lesions (WMLs) was rated using the Fazekas scale based on magnetic resonance imaging analysis. White matter structure was quantified using the voxel-based morphometry method. The correlations between NLRP3 levels, NfL levels, neuropsychological dysfunction, the degree of WMLs, and white matter volume (WMV) were analyzed in alcohol dependence patients.

**Results:** Serum NLRP3 and NfL levels were higher in the alcohol dependence group. NLRP3 levels were irrelevant to monthly alcohol assumption as well as to the MoCA, PSQI, GAD-7, PHQ-9, and Fazekas scale scores and WMV. NfL levels were positively correlated with the PSQI and PHQ-9 scores as well as the degree of WMLs and negatively correlated with the MoCA scores and WMV. No associations were evident between NfL and monthly alcohol assumption and GAD-7 scores in the alcohol dependence group.

**Conclusion:** This study supports the potential value of serum NfL as a non-invasive biomarker in alcohol dependence. The association with neuropsychological dysfunction and degree of WMLs has implications to use NfL as a promising biomarker to assess the severity of brain damage as well as the progression and prognosis of alcohol dependence.

## Introduction

Alcohol dependence is a common global public health problem. Long-term alcohol abuse can result in neuroinflammation, oxidative stress, as well as structural and functional disorders of the brain (1). Structural changes, including myelin disruptions, white matter lesions (WMLs) (2), the atrophy of gray and white matter accompanied by sulcal widening and ventricular enlargement (3), and functional disorders such as cognitive impairment and psychological abnormality are commonly seen in alcohol dependence (4, 5).

A previous study has reported that alcohol dependence is related to increased WMLs. Among middle-aged men, excessive drinking may be associated with both microstructural and macrostructural white matter injury (6). White matter are susceptible to the neurotoxic effects of alcohol caused by thiamine deficiency (7). The Fazekas scale is a widely used scale to evaluate the severity of WMLs in both periventricular (PV) and deep white matter (DWM) areas.

Nucleotide-binding oligomerization domain containing 3 (NLRP3) is one of the extensively studied inflammasomes that acts as a sensor of metabolic stress and plays a vital role in the neuroinflammation and demyelination processes of several neurological diseases. Previous studies have demonstrated that alcohol can activate the NLRP3-inflammasome complex by stimulating the activation of caspase-1 and induction of IL-1β and IL-18 pro-inflammatory cytokines. NLRP3 is increased in the cultured astroglial cells, microglial cells, and cerebral cortex of mice with chronic alcohol treatment (8).

Neurofilaments, which consist of heteropolymers of three subunits named neurofilament light chain (NfL), neurofilament medium chain (NfM), and neurofilament heavy chain (NfH), are important elements of cytoskeletal protein, and they provide structural support for neurons. Following axon damage, neurofilaments are released into the extracellular space, followed by the cerebrospinal fluid (CSF) and blood. Therefore, NfL can be detected in the CSF and plasma or serum after neurological injury, and it is regarded as a potential biomarker of axonal and neuron damage (9, 10). Recent studies have indicated that NfL expression changes in various neurological diseases, for which it is considered to be a promising marker. It may not only facilitate accurate diagnosis but also predict disease progression and assess treatment responses (11). NfL is associated with the severity of white matter hyperintensities and brain atrophy (12).

Voxel-based morphometry (VBM) is a comprehensive and objective automatic whole-brain analysis method that allows the detection of subtle morphometric differences in the brain structure (13). The atrophy of white matter is commonly seen in alcohol dependence (14). In the present study, we analyzed white matter volume (WMV) in patients with alcohol dependence using VBM.

To explore the potential biomarker for alcohol dependence, we first detected the expression of NLRP3 and NfL in alcohol dependence patients' serum and found that NLRP3 and NfL levels were higher in alcohol dependence patients than in controls. Furthermore, we analyzed the correlations between NLRP3/NfL levels, alcohol consumption, the severity of neuropsychological dysfunction, WMLs, and WMV. We demonstrated the NfL levels were closely correlated with the MoCA scores, PSQI scores, PHQ-9 scores, Fazekas scale scores, and WMV in alcohol dependence patients, which suggests that NfL may act as a biomarker for monitoring disease progression or predicting prognosis in alcohol dependence.

## Materials and Methods

### Participants

We enrolled 50 patients with alcohol dependence and 50 age- and sex-matched controls with no drinking history, which were set as the alcohol dependence group and control group, respectively. The diagnosis of alcohol dependence was based on the standards of the fourth edition of the Diagnostic and Statistical Manual of Mental Disorders.

The criteria for the diagnosis of alcohol dependence were as follows (15): tolerance to alcohol, withdrawal syndrome, greater alcohol use than intended, desire to use alcohol and inability to control use, devotion of a large proportion of time to getting and using alcohol, recovering from alcohol use, neglect of social, work, or recreational activities, and continued alcohol use despite physical or psychological problems. Alcohol dependence was designated based on the fulfillment of three or more of the aforementioned criteria within 12 months. The exclusion criteria for both groups were as follows: (1) acute phase of alcohol withdrawal syndrome or Wernicke-Korsakoff syndrome; (2) history of drug abuse or other substance dependence such as cocaine, methamphetamine, marijuana, nicotine, and cigarettes; (3) history of other significant neurological or psychological diseases; (4) history of psychological diseases such as depression and anxiety; (5) history of severe brain trauma or cranial surgery; and (6) medically uncontrolled chronic hypertension and diabetes mellitus, hyperlipidemia chronic obstructive pulmonary disease, and human immunodeficiency virus (HIV)/acquired immune deficiency syndrome (AIDS).

We collected the participants' clinical data, including age, sex, previous history, alcohol use (frequency, quantity, and years of alcohol consumption), MRI results, and neuropsychological assessment outcomes, including the Montreal cognitive assessment (MoCA), Pittsburgh sleep quality index (PSQI), generalized anxiety disorder (GAD-7), and patient health questionnaire-9 (PHQ-9) scores. Alcohol use quantity was measured by standard drinks. A standard drink was defined as 12 oz. beer, 5 oz. wine, or 1.5 oz. liquor. The alcohol dependence scale (ADS) was used to measure the severity of the participant's dependence on alcohol. The study was approved by the Ethics Committee of the First Affiliated Hospital of Zhengzhou University (2018-KY-91). All the participants or their guardians provided written informed consent.

### Magnetic Resonance Imaging Data

MRI scans were performed using a 3.0 T scanner (Philips Healthcare, Amsterdam, Netherlands). Sagittal and axial T1-weighted images, T2-weighted images, axial and sagittal fast fluid-attenuated inversion recovery (FLAIR) images, axial diffusion, and apparent diffusion coefficient mapped images were analyzed.

### Fazekas Scale

**Figure 1:**
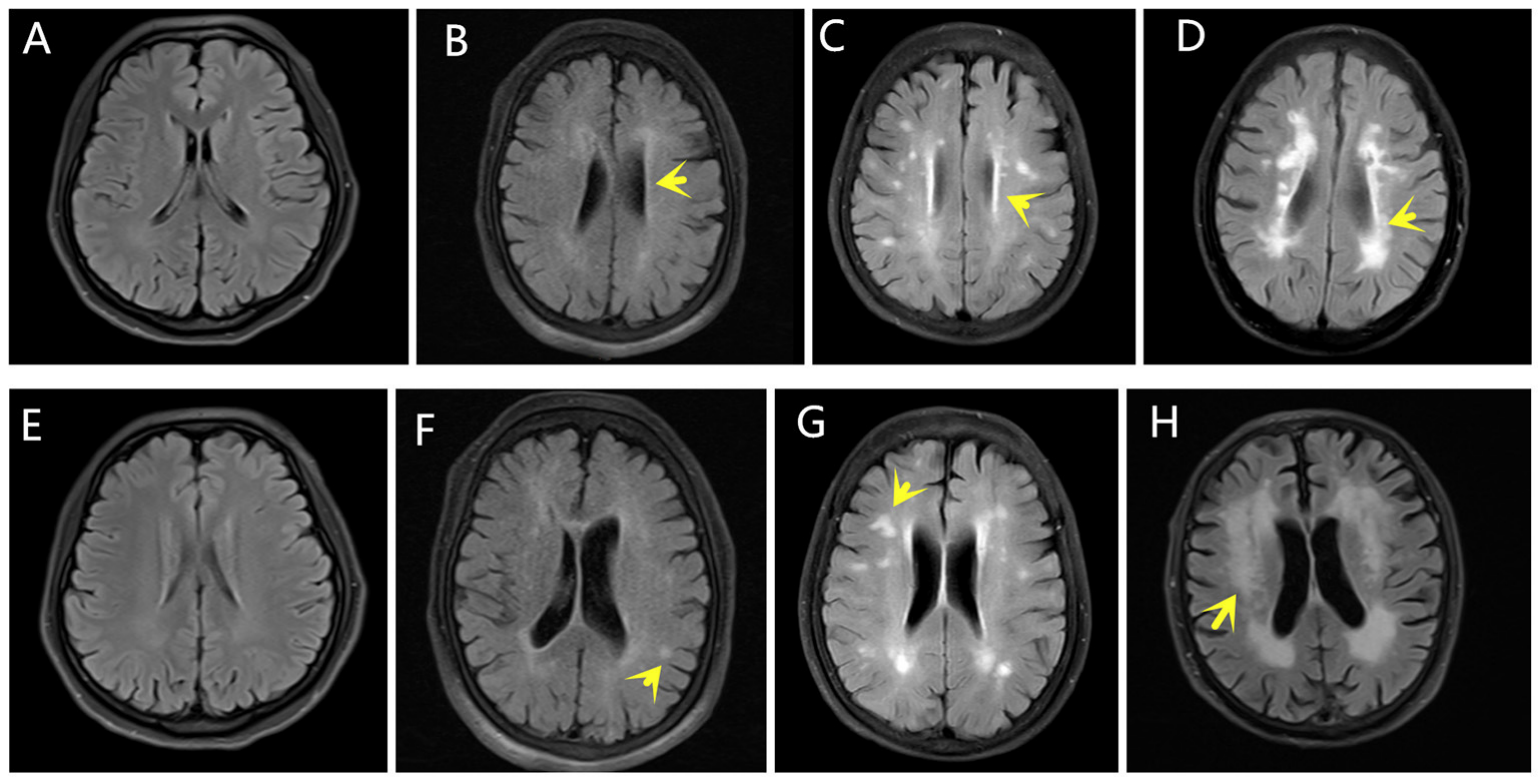
Fazekas scores. The yellow arrows indicate WMLs on FLAIR MRI. The PV scores were categorized as follows: **(A)** 0, **(B)** 1, **(C)** 2, **(D)** 3. The DWM scores were categorized as follows: **(E)** 0, **(F)** 1, **(G)** 2, **(H)** 3.

The degree of WMLs was rated using the Fazekas scale during MRI analysis. PV and DWM demyelination were scored separately using the four-point scale according to the Fazekas scale on the FLAIR MRI data for the axial plane. The PV scores were categorized as follows: 0 (absence of white matter signal abnormalities), 1 (caps or pencil-thin lining), 2 (smooth halo), or 3 (irregular PV lesions extending into the DWM). The DWM scores were categorized as follows: 0 (absence of white matter signal abnormalities), 1 (punctate foci), 2 (beginning confluent white matter signal abnormalities), or 3 (white matter signal abnormalities in large confluence areas) (16). The Fazekas scale scores were equal to the sum of the PV and DWM scores. The Fazekas scale assessments were completed by an experienced neuroradiologist blinded to the clinical data. Figure 1 shows the rating of the Fazekas scores in the MRI FLAIR scans for the PV and DWM areas.

### VBM Analysis

Brain T1-weighted images were taken on Siemens Magnetom Skyra 3.0 T scanners. T1-weighted images were processed with pipeline steps using the CAT12 toolbox (http://dbm.neuro.uni-jena.de/cat12/). All images were checked and processed with the standard setting for all steps, including segmentation into gray and white matter and cerebrospinal fluid, bias correction, normalization into Montreal Neurological Institute space, and non-linear modulation. Then, the images were resampled to a volume image resolution of 1.5 × 1.5 × 1.5 mm^3^. Finally, the images were smoothed with a Gaussian kernel of 6 mm full width at half maximum. The total intracranial volume (TIV) of each participant was also calculated for the next comparative analysis (17, 18).

### Serum Collection

Fresh blood samples were obtained by venipuncture and collected into non-anticoagulant tubes. The serum was obtained by the centrifugation of blood samples at 200 × g for 20 min at 4°C. The supernatants were collected in 1.5 ml polypropylene tubes at −80°C.

### Enzyme-Linked Immunosorbent Assay

NLRP3 levels were detected using an ELISA kit (CUSABIO). An antibody specific for NLRP3 was pre-coated onto a microplate. Standards and samples were pipetted into the wells and any NLRP3 present was bound by the immobilized antibody. After removing any unbound substances, a biotin-conjugated antibody specific for NLRP3 was added into the wells. After washing, avidin conjugated horseradish peroxidase was added into the wells. Following a wash to remove the unbound avidin-enzyme reagent, a substrate solution was added into the wells and color developed in proportion to the amount of NLRP3 bound in the initial step. The color development was stopped and the intensity of the color was measured.

### Neurofilament Detection

The concentration of NfL was detected using single-molecule array (Simoa; Quanterix, USA), as previously described in detail (19). All the measurements were taken by laboratory technicians blinded to the clinical data.

### Statistical Analysis

Data were presented as mean ± standard deviation (SD) if the continuous variables were distributed normally according to the Kolmogorov–Smirnov test. Otherwise, data were presented as median [interquartile range (IQR)]. To analyze the differences, the normally distributed data were compared between the groups using the Student's *t*-test. The categorical data were compared using the chi-square test and abnormal distribution data were compared using the Wilcoxon test. Analysis of covariance was used to evaluate the difference in WMV between the groups and TIV was set as the covariate. The correlation analysis was performed using the Spearman correlation. TIV was set as the covariate and partial correlation analysis was used to evaluate the relationships between NLRP3 levels, NfL levels, and WMV. The differences were considered to be significant when the *P*-values were <0.05. All the analyses were performed using IBM SPSS Statistics 21. The diagram was generated with GraphPad Prism 5.

## Results

### Clinical Data

We enrolled 50 patients with alcohol dependence and 50 controls. The alcohol dependence group comprised 36 men and 14 women, ranging from 30 to 65 years of age (mean = 48.8 years), and the controls comprised 35 men and 15 women, ranging from 31 to 62 years of age (mean = 48.7 years). The mean years of schooling of the alcohol dependence patients and controls were 9.4 ± 3.3 and 10.2 ± 3.2, respectively. There were no significant differences in the age, sex, and years of schooling between the alcohol dependence group and controls.

The years of alcohol consumption of the alcohol dependence group ranged from 5 to 50 years. The mean drinking frequency was 4.8 times per week. The drinks/per drinking day were 6.7 ± 3.7. The drinks/per drinking day were 7.7 ± 3.7 in men and 4.3 ± 2.1 in women (*P* < 0.01). The drinking modalities were almost the same on every drinking day. The median quantity of alcohol consumption was 112.9 standard drinks per month in the last year. The ADS scores of the alcohol dependence group were 16.24 ± 5.92.

The MoCA scores were lower in the alcohol dependence group than in the controls and the PSQI, GAD-7, and PHQ-9 scores were higher in the alcohol dependence group than in the controls. There were statistical differences in the MoCA, PSQI, GAD-7, and PHQ-9 scores between the alcohol dependence group and controls (*P* < 0.05).

The Fazekas scale scores, PV scores, and DWM scores were higher in the alcohol dependence group than in the controls. There were significant differences in the Fazekas scale scores, PV scores, and DWM scores between the alcohol dependence group and controls (*P* < 0.05), which suggest that the WMLs were more severe in the alcohol dependence group than in the controls. WMV was 516.94 ± 45.40 cm^3^ in the alcohol dependence group and 531.51 ± 42.79 cm^3^ in the controls. After adjustment with the TIV covariate, mean WMV was 512.98 cm^3^ in the alcohol dependence group and 534.73 cm^3^ in the controls. The WMV of alcohol dependence patients was significantly less than that of the controls (*P* < 0.05, Table 1).

**Table 1:** Clinical data of alcohol dependence patients and controls.

	**Alcohol dependence group**	**Controls**	***P*-value**
Male, n (%)	36 (72)	35 (70)	0.826
Age, y, mean ± SD	48.8 ± 9.5	48.7 ± 6.9	0.189
Years of schooling, y, mean ± SD	9.4 ± 3.3	10.2 ± 3.2	0.587
Hypertension, n (%)	9 (18)	7 (14)	0.585
Diabetes mellitus, n (%)	4 (8)	4 (8)	1.000
Hyperlipidemia, n (%)	6 (12)	5 (10)	0.749
MoCA scores, mean ± SD	23.26 ± 4.84	27.34 ± 1.88	0.000^*^
PSQI scores, median (IQR)	6 (3, 9)	2.00 (1.00, 4.25)	0.000^*^
GAD-7 scores, median (IQR)	3.00 (0.75, 7.25)	2.00 (1.00, 2.25)	0.048^*^
PHQ-9 scores, median (IQR)	5 (3, 10)	2.00 (1.00, 4.25)	0.000^*^
Fazekas scale scores, median (IQR)	2.00 (1.0, 3.25)	1 (0, 2)	0.000^*^
PV scores, median (IQR)	1 (1, 2)	1 (0, 1)	0.000^*^
DWM scores, median (IQR)	1 (0, 2)	0.5 (0, 1)	0.006^*^
^a^WMV (cm^3^), mean ± SD	516.94 ± 45.40	531.51 ± 42.79	0.102
^b^Adjusted WMV(cm^3^), mean	512.98	534.73	0.000^*^

*Data are presented as mean ± standard deviation, number (percentage), or median (interquartile range)*.

a *The actual WMV data without TIV as a covariate*.

b *Covariates appearing in the model are evaluated at the following values: TIV = 1,458.01*.

* *P < 0.05*.

### Expression of Serum NLRP3 and NfL in Alcohol Dependence Patients

**Figure 2:**
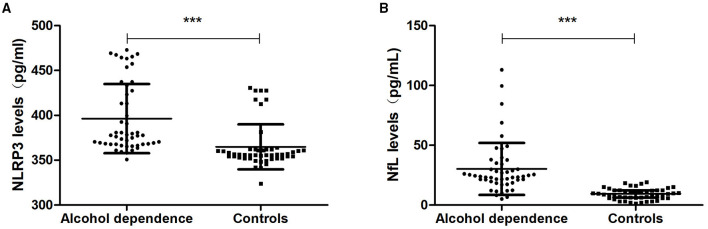
NLRP3 and NfL levels in the alcohol dependence group and the controls. **(A)** The NLRP3 levels in the alcohol dependence group were higher than in the controls (*P* < 0.001). **(B)** The NfL levels in the alcohol dependence group were higher than in the controls (*P* < 0.001). ***P < 0.001.

The expression levels of serum NLRP3 were 377.72 (367.73, 428.90) pg/ml in the alcohol dependence group and 356.35 (352.68, 361.92) pg/ml in the controls. The expression levels of serum NLRP3 in the alcohol dependence group were higher than those in the controls (*P* = 0.000) (Figure 2A).

The expression levels of serum NfL were 23.86 (19.39, 34.43) pg/ml in the alcohol dependence group and 9.42 (6.13, 12.25) pg/mL in the controls. The expression levels of serum NfL in the alcohol dependence group were higher than those in the controls (*P* = 0.000) (Figure 2B).

### Relationships Between Serum NLRP3 Levels and Alcohol Consumption, Neuropsychological Function, and Severity of WMLs

To determine whether NLRP3 levels were associated with alcohol assumption, we analyzed the correlation between serum NLRP3 levels and monthly alcohol assumption in the last year. The results indicated that NLRP3 levels were irrelevant to monthly alcohol assumption in the alcohol dependence group (*P* > 0.05).

To explore whether NLRP3 expression could reflect the level of neuropsychological function, we analyzed the correlation between NLRP3 levels and the MoCA, PSQI, GAD-7, and PHQ-9 scores. In the alcohol dependence group, NLRP3 levels were irrelevant to the MoCA, PSQI, GAD-7, and PHQ-9 scores (*P* > 0.05).

**Figure 3:**
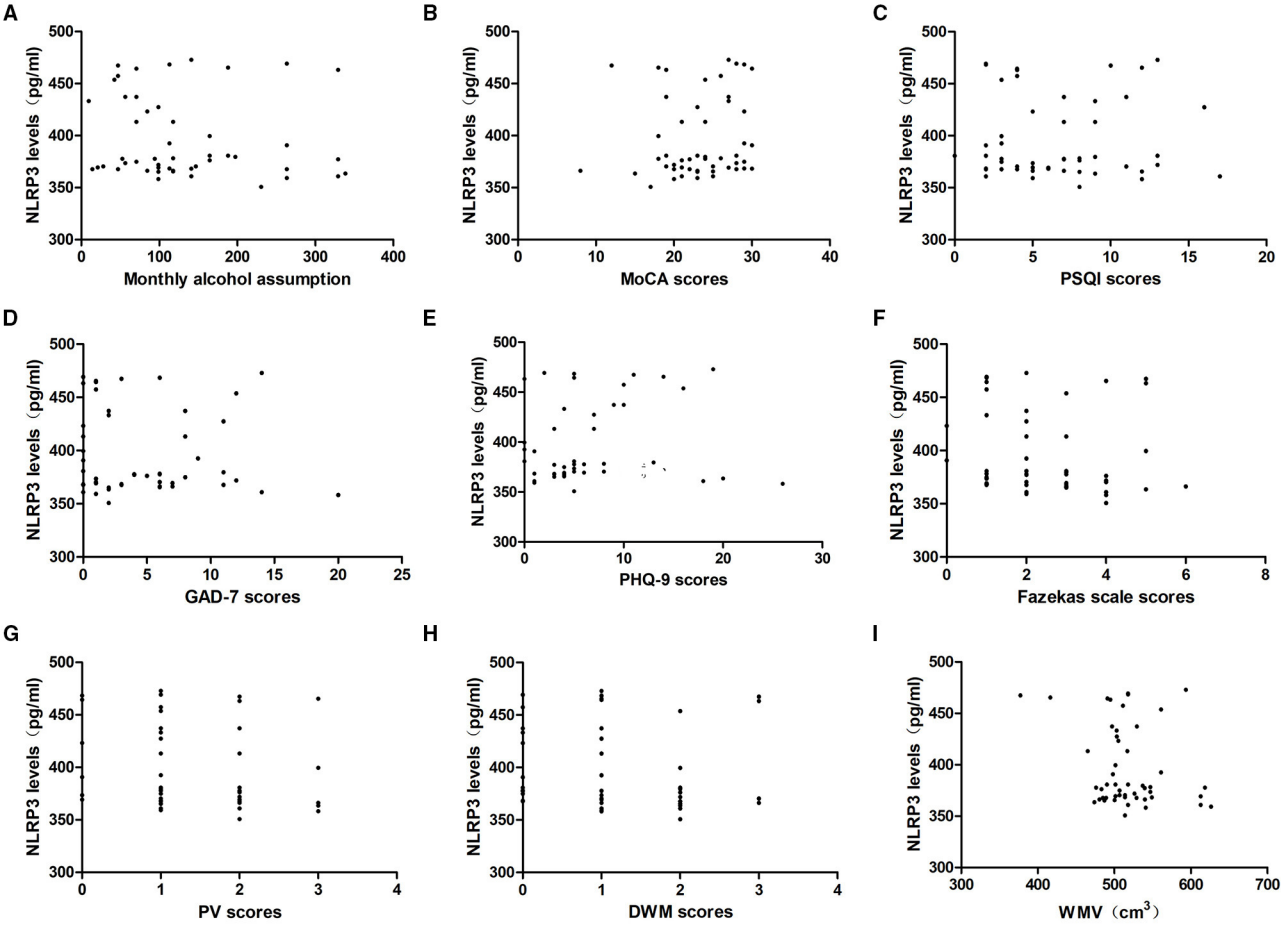
Relationship between serum NLRP3 levels and alcohol assumption, neuropsychological function, the severity of WMLs, and WMV in the alcohol dependence group. Correlation of serum NLRP3 levels with **(A)** monthly alcohol consumption, **(B)** MoCA scores, **(C)** PSQI scores, **(D)** GAD-7 scores, **(E)** PHQ-9 scores, **(F)** Fazekas scale scores, **(G)** PV scores and **(H)** DWM scores, **(I)** WMV. NLRP3 levels were irrelevant to monthly alcohol consumption, MoCA scores, PSQI scores, GAD-7 scores, PHQ-9 scores, Fazekas scale scores, PV scores, DWM scores, and WMV in the alcohol dependence group (*P* > 0.05).

MRI images were analyzed and the degree of WMLs was rated using the Fazekas scale. WMV was measured by VBM. In the alcohol dependence group, NLRP3 levels were irrelevant to the Fazekas scale scores, PV scores, DWM scores, and WMV (*P* > 0.05, Figure 3).

In the controls, NLRP3 levels were irrelevant to the MoCA, PSQI, GAD-7, and PHQ-9 scores as well as the Fazekas scale scores, PV scores, DWM scores, and WMV (*P* > 0.05).

### Relationships Between Serum NfL Levels and Alcohol Consumption, Neuropsychological Function, and Severity of WMLs

To determine whether NfL levels were associated with alcohol assumption, we analyzed the correlation between serum NfL levels and monthly alcohol assumption in the last year. The results indicated that NfL levels were irrelevant to monthly alcohol assumption (*P* > 0.05).

**Figure 4:**
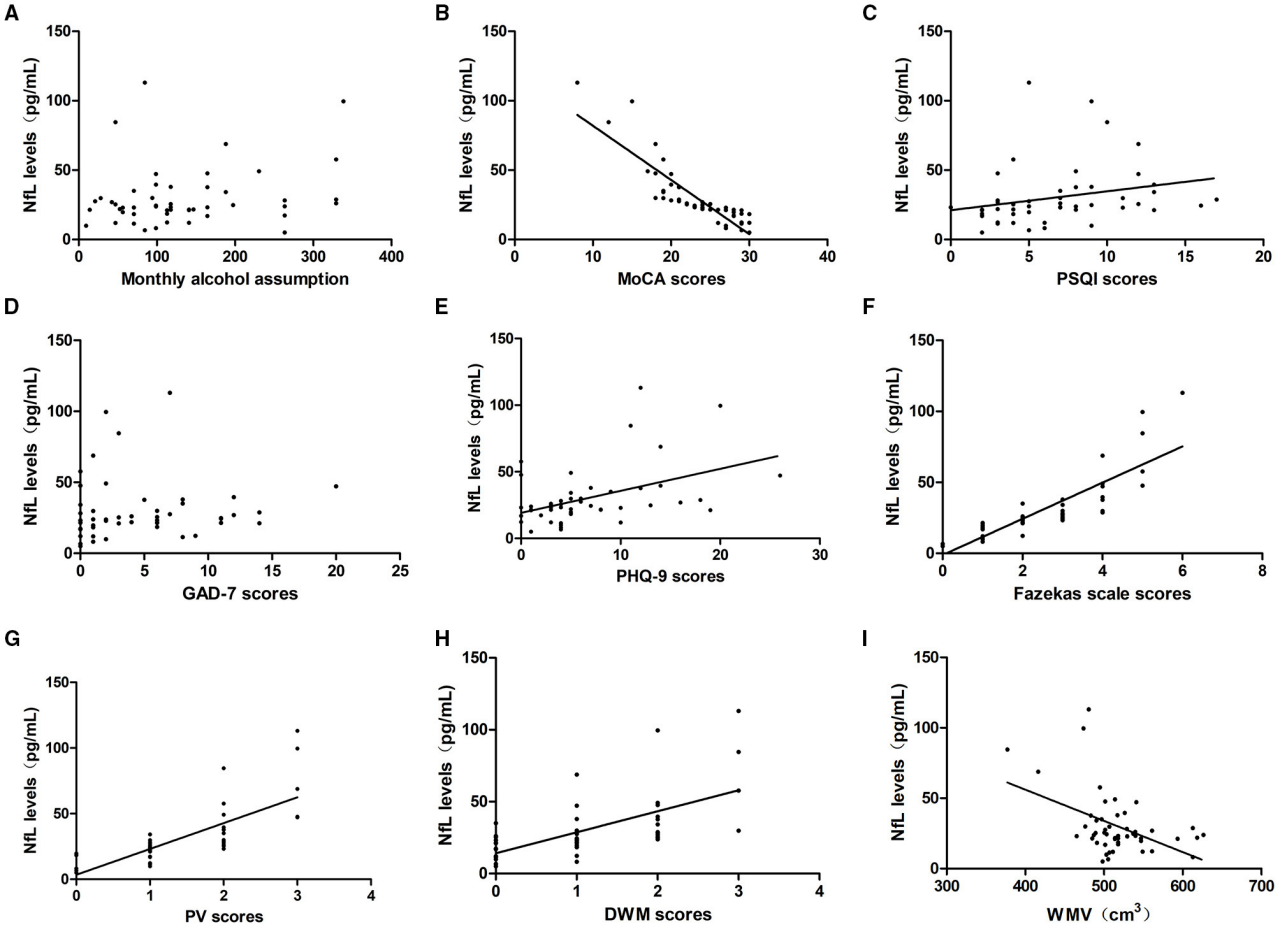
Relationship between serum NfL levels and alcohol assumption, neuropsychological function, the severity of WMLs, and WMV in the alcohol dependence group. Correlation of serum NfL levels with **(A)** monthly alcohol consumption, **(B)** MoCA scores, **(C)** PSQI scores, **(D)** GAD-7 scores, **(E)** PHQ-9 scores, **(F)** Fazekas scale scores, **(G)** PV scores and **(H)** DWM scores, **(I)** GMV. NfL levels were irrelevant to monthly alcohol assumption and GAD-7 scores (*P* > 0.05). NfL levels were negatively correlated with MoCA scores and WMV, and positively correlated with PSQI scores, PHQ-9 scores, Fazekas scale scores, PV scores, and DWM scores (*P* < 0.05).

To explore whether NfL expression could reflect the level of neuropsychological function, we analyzed the correlation between NfL levels and the MoCA, PSQI, GAD-7, and PHQ-9 scores. In the alcohol dependence group, NfL levels were negatively correlated with the MoCA scores (*r* = −0.94, *P* = 0.000) and positively correlated with the PSQI scores (*r* = 0.461, *P* = 0.001) and PHQ-9 scores (*r* = 0.423, *P* = 0.002). However, they were not associated with the GAD-7 scores (*r* = 0.218, *P* = 0.128). The NfL levels were positively correlated with the Fazekas scale scores (*r* = 0.930, *P* = 0.000), PV scores (*r* = 0.815, *P* = 0.000), and DWM scores (*r* = 0.689, *P* = 0.000), but negatively correlated with WMV (*r* = −0.343, *P* = 0.015). When TIV was set as a covariate, the partial correlation analysis indicated that NfL levels were still negatively correlated with WMV (*r* = −0.514, *P* = 0.000, Table 2; Figure 4).

**Table 2:** Relevance analysis of serum NfL levels and alcohol assumption, neuropsychological function, the severity of WMLs, and WMV in the alcohol dependence group.

		***r***	***P*-value**
NfL levels, median (IQR) pg/ml	23.86 (19.39, 34.43)		
Alcohol consumption per month, median (IQR) standard drinks	112.9 (70.5, 170.475)	0.2038	0.1558
MoCA scores, mean ± SD	23.26 ± 4.84	−0.94	0.000^*^
PSQI scores, median (IQR)	6 (3, 9)	0.461	0.001^*^
GAD-7 scores, median (IQR)	3.00 (0.75, 7.25)	0.218	0.128
PHQ-9 scores, median (IQR)	5 (3, 10)	0.423	0.002^*^
Fazekas scale scores, median (IQR)	2.00 (1.0, 3.25)	0.930	0.000^*^
PV scores, median (IQR)	1 (1, 2)	0.815	0.000^*^
DWM scores, median (IQR)	1 (0, 2)	0.689	0.000^*^
WMV (cm^3^), mean ± SD	516.94 ± 45.40	−0.343	0.015

*Data are presented as mean ± standard deviation, or median (interquartile range). The Spearman analysis indicated the relationship between serum NfL levels and alcohol assumption, neuropsychological function, the severity of WMLs, and WMV in the alcohol dependence group*.

* *P < 0.05*.

In the control group, NfL levels were irrelevant to the MoCA, PSQI, GAD-7, and PHQ-9 scores as well as the Fazekas scale scores, PV scores, DWM scores, and WMV (*P* > 0.05).

### ROC Curve for NLRP3 and NfL Predicts Alcohol Dependence

**Figure 5:**
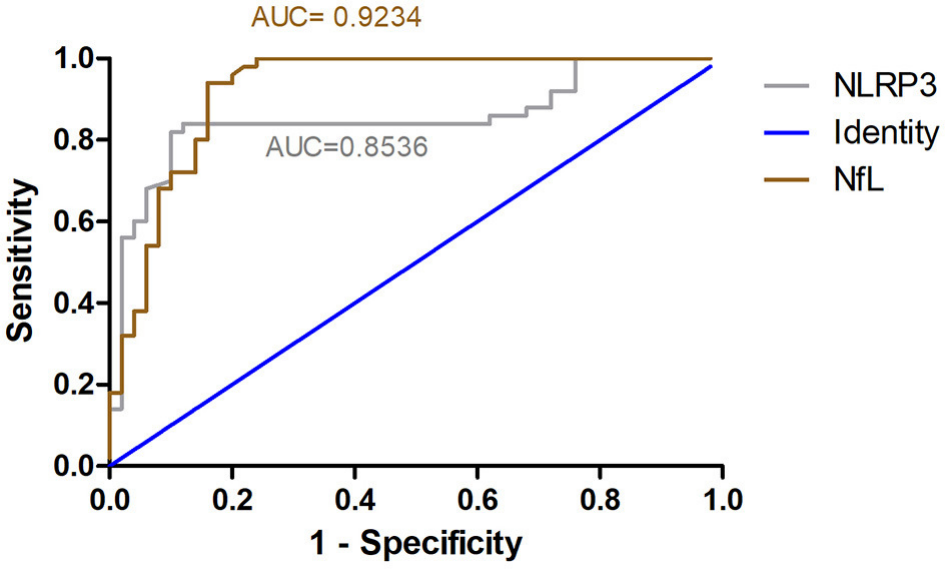
ROC curve for NLRP3 and NfL predicts alcohol dependence. ROC curve analysis was used to evaluate the predictive value of NLRP3 and NfL for the diagnosis of alcohol dependence. The areas under the ROC curve were 0.8536 for NLRP3 levels and 0.9234 for NfL levels.

ROC curve analysis was used to evaluate the predictive value of NLRP3 and NfL for the diagnosis of alcohol dependence. The areas under the ROC curve were 0.8536 (95% CI = 0.7727–0.9345, *P* < 0.01) for NLRP3 levels and 0.9234 (95% CI = 0.8682–0.9786, *P* < 0.01) for NfL levels (Figure 5). At an NLRP3 cut-off of 364.6 pg/ml, the sensitivity for predicting alcohol dependence was 84% and the specificity was 88%. At an NfL cut-off of 16.65 pg/ml, the sensitivity for predicting alcohol dependence was 84% and the specificity was 94%.

## Discussion

With increasing alcohol consumption, alcohol use disorder has become an intractable and public concern globally. Alcohol use disorder consists of alcohol dependence, alcohol abuse, and dependence or harmful use. Alcohol dependence is characterized by compulsive alcohol consumption or a shift from drinking for pleasure to compulsive alcohol-seeking behavior. Several alcohol dependence patients who drink heavily have cognitive disorders such as mild cognitive impairment, temporary cognitive deficits, and dementia. Prospective studies have indicated that alcohol dependence patients have more than double the risk of later severe memory injury than controls (20, 21). Psychiatric disorders are common in alcohol dependence patients, including depression, anxiety, sleep disturbance, suicide, and the abuse of other drugs and substances. Approximately 40% of patients with alcohol dependence have psychological dysfunction. Alcohol dependence and psychiatric disorders worsen with each other.

Our study evaluated the cognitive and psychological conditions of alcohol dependence patients using the MoCA, PSQI, GAD-7, and PHQ-9 assessments. The results indicated that the MoCA scores in the alcohol dependence group were lower than those in the controls, which showed that enrolled alcohol dependence patients already had cognitive impairments, consistent with alcohol's propensity to cause cognitive injury. The study found that the PSQI, GAD-7, and PHQ-9 scores in the alcohol dependence group were higher than those in the controls, which suggests that alcohol dependence patients tend to have more trouble with sleep and psychiatric disorders than non-alcohol controls.

Previous studies have identified the harmful effects of alcohol dependence on the brain structure, including WMLs and the atrophy of gray and white matter. There is a U-shaped relationship between alcohol dose and WMLs; moderate drinking may be associated with better white matter health, whereas drinking beyond recommended guidelines may be associated with more white matter damage (7). We assessed the severity of WMLs using the Fazekas scale and found that the Fazekas scale scores were higher in the alcohol dependence group than in the controls, which indicated that WMLs were more severe in alcohol dependence patients than in controls. There was no U-shaped trend between the Fazekas scale scores and alcohol consumption in the alcohol dependence group. We did not see any improvement in WMLs with an increase in alcohol consumption in the alcohol dependence group, perhaps because the enrolled alcohol dependence patients' tended to have a history of long-term heavy alcohol use beyond the recommended dose. Previous studies have shown that WMV is significantly smaller in patients with alcohol dependence than in healthy controls (13, 22). In the present study, we also obtained a similar result involving WMV in alcohol dependence patients.

Several alcohol dependence patients are difficult to diagnose initially, since they are likely to have a normal life and family life, go to work regularly, and have general complaints like most healthy people such as depression, anxiety, insomnia, dreaminess, and fatigue. Therefore, we aimed to discover a biomarker for the assessment of the clinical progression and prognosis of alcohol dependence.

The association of NLRP3 has been widely explored in chronic alcoholism. Previous studies have identified alcohol as able to activate the NLRP3-inflammasome complex by stimulating the activation of caspase-1 and induction of IL-1β and IL-18 pro-inflammatory cytokines. An increase in NLRP3 is observed in the cultured astroglial cells, microglial cells, and cerebral cortex of mice with chronic alcohol treatment (8). Thus, we speculated whether NLRP3 changed in the serum of alcohol dependence patients and could act as a biomarker. The study found that NLRP3 levels were higher in the alcohol dependence group than in the controls, but that there were no associations between NLRP3 levels and alcohol consumption, MoCA scores, PSQI scores, GAD-7 scores, PHQ-9 scores, or Fazekas scale scores. This suggests that although NLRP3 was increased in alcohol dependence patients, it may not be a sensitive marker for assessing the severity of brain damage and prognosing alcohol dependence.

NfL is released into the extracellular space, followed by the CSF and blood after axonal damage; therefore, NfL can be detected in the CSF and serum or plasma after neurological injury (23). The pattern of NfL changes is almost identical in the serum and CSF (24). Further, serum/plasma NfL detection is a non-invasive and more direct method than lumbar puncture. In the study, we explored the possibility of NfL as a potential non-invasive and sensitive biomarker for monitoring the progression and prognosis of alcohol dependence. Simoa, a newly developed ultrasensitive immunoassay, is by far the most sensitive platform for detecting the concentration of NfL and it provides a better agreement between the CSF and serum NfL than other analytical platforms. Therefore, we employed Simoa to detect the expression of serum NfL.

Our study first detected the expression of NfL in alcohol dependence patients and found that the expression levels of serum NfL were increased in alcohol dependence patients than in age- and sex-matched controls. As shown in the scatterplot in the study, there was a negative correlation between the MoCA scores and NfL levels and positive correlations between the PSQI scores, PHQ-9 scores, and NfL levels in alcohol dependence patients. Moreover, we found that NfL levels were positively correlated with the degree of WMLs and negatively with WMV. The Fazekas scale scores were higher and WMV was smaller in the alcohol dependence group than in the controls. The quantitative reports identified that the number of neurons and weight of brain tissue in alcohol dependence patients were decreased. A previous study indicated that NfL levels are related to the progression of brain atrophy and WMLs (25). Further, NfL is generally recognized as a potential biomarker of axonal and neuron damage, which may explain why NfL levels reflect the degree of WMLs and WMV.

Taken together, the expression levels of NfL were speculated to reflect the severity of WMLs and neuropsychological impairment in alcohol dependence. However, the relationship between NfL levels and alcohol dependence requires further validation with additional samples, and longitudinal data on NfL levels in the serum and disease progression are not yet available.

## Conclusion

Our study confirmed that the expression of NLRP3 and NfL was higher in alcohol dependence patients than in controls. NfL levels were negatively correlated with the MoCA scores and WMV, and positively correlated with the PSQI scores, PHQ-9 scores, and degree of WMLs, suggesting that the change in the expression of NfL may reflect the progression and prognosis of alcohol dependence.

## Data Availability Statement

The original contributions presented in the study are included in the article/supplementary material, further inquiries can be directed to the corresponding author.

## Ethics Statement

The study was approved by the Ethics Committee of First Affiliated Hospital of Zhengzhou University (2018-KY-91). The patients/participants provided their written informed consent to participate in this study.

## Permission

The study state that the permission of MoCA scale has been obtained.

## Author Contributions

YL: methodology, formal analysis, data curation, writing–original draft, and writing-review and editing. RD: investigation and writing–review and editing. ZG: methodology, investigation, and writing-review and editing. LJ and YZ: data curation. TZ: formal analysis. YJ: conceptualization, methodology, supervision, and funding acquisition. The first draft of the manuscript was written by YL and all authors agree to be accountable for the content of the work.

## Funding

This study was funded by National Key R&D Program of China (2018YFC1314400 and 2018YFC1314403).

## Conflict of Interest

The authors declare that the research was conducted in the absence of any commercial or financial relationships that could be construed as a potential conflict of interest.

## Publisher's Note

All claims expressed in this article are solely those of the authors and do not necessarily represent those of their affiliated organizations, or those of the publisher, the editors and the reviewers. Any product that may be evaluated in this article, or claim that may be made by its manufacturer, is not guaranteed or endorsed by the publisher.

## Abbreviations

ELSA: enzyme-linked immunosorbent assay
NLRP3: nucleotide-binding oligomerization domain containing 3
Simoa: Single-Molecule Array
NfL: neurofilament light
MoCA: Montreal cognitive assessment
PSQI: Pittsburgh sleep quality index
GAD-7: generalized anxiety disorder
PHQ-9: patient health questionnaire-9
WMLs: white matter lesions
MRI: magnetic resonance imaging
FLAIR: fluid-attenuated inversion recovery
VBM: voxel-based morphometry
NfM: neurofilament medium chain
NfH: neurofilament heavy chain
CSF: cerebrospinal fluid
GMV: gray matter volume
WMV: white matter volume
PV: periventricular
DWM: deep white matter
HIV: human immunodeficiency virus
AIDS: acquired immune deficiency syndrome
FWHM: full width at half maximum
TIV: total intracranial volume
HRP: Horseradish Peroxidase.
